# Supplementary material for: Warming induces unexpectedly high soil respiration in a wet tropical forest
Source: Nat Commun. 2025 Sep 16;16:8222. doi: 10.1038/s41467-025-62065-6 (PMC12441113; doi:10.1038/s41467-025-62065-6)
Supplement: Supplementary file 1 — Supplementary Information [file 41467_2025_62065_MOESM1_ESM.pdf]

Supplemental Figures, Tables, Materials and Methods: Warming induces unexpectedly high soil respiration in a wet tropical forest

Tana E. Wood[1]*; Colin Tucker[2,3]; Aura M. Alonso-Rodríguez[4,5]; M. Isabel Loza[6]; Iana F. Grullón-Penkova[1]; Molly A. Cavaleri[7]; Christine S. O'Connell[8,9]; Sasha C. Reed[2]

1 USDA Forest Service International Institute of Tropical Forestry, Río Piedras, Puerto Rico, 00926, USA

2 US Geological Survey, Southwest Biological Science Center, Moab, Utah, 84532, USA

3 USDA Forest Service Northern Research Station, Houghton, Michigan, 49931, USA

4 Gund Institute for Environment, University of Vermont, Burlington, Vermont, USA

5 Rubenstein School of Environment and Natural Resources, University of Vermont, Burlington,

Vermont, USA

6 Center for Tree Science, Morton Arboretum, Lisle, IL 60532 USA

7 College of Forest Resources and Environmental Science, Michigan Technological University, Houghton, Michigan, 49931, USA.

8 Department of Environmental Studies, Macalester College, St. Paul, Minnesota, 55105, USA.

9 Biology Program, Schmid College of Science and Technology, Chapman University, Orange, California 92866 USA

Included within:

- Supplemental Figures
    - Supplemental Figure S1. Mean fine live root biomass prior to warming (March 2016) and 6 months after warming was initiated (March 2017) in control (blue) and warmed (red) plots.
    - Supplemental Figure S2. Spatial interpolation of the mean soil CO2 fluxes across the study site.
    - Supplemental Figure S3. Pre-warming soil respiration rates by plot.
    - Supplemental Figure S4. Interactive effects of Soil moisture and Temperature on Soil Respiration.
    - Supplemental Figure S5. Diurnal mean temperature versus diurnal mean respiration rate by topographic position and treatment presented on the same scale.
- Supplemental Tables
    - Supplemental Table S1. Generalized least squares model regression coefficients; model results with and without upper slope plots with extreme values.
    - Supplemental Table S2. Generalized least squares model regression coefficients.
- Supplemental Materials and Methods
    - A. Instrument Configuration and Data Processing
    - B. Validating notably high soil CO2 fluxes
    - C. Generalized least squares / linear mixed effects models

Any use of trade, firm, or product names is for descriptive purposes only and does not imply endorsement by the U.S. Government

Supplemental Figures

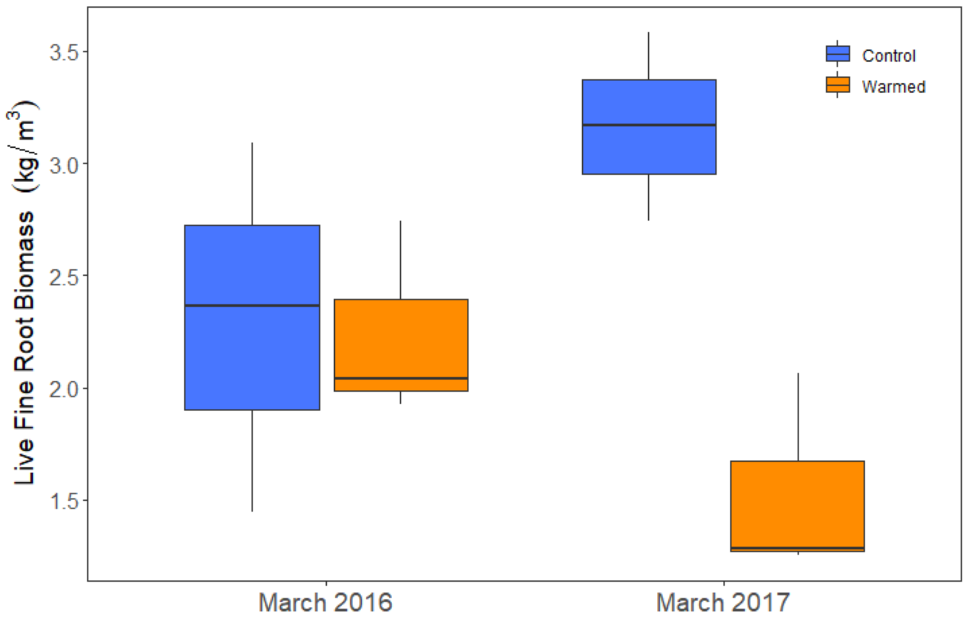

Supplemental Figure S1. Mean fine live root biomass prior to warming (March 2016) and 6 months after warming was initiated (March 2017) in control (blue) and warmed (red) plots. Difference between treatments was analyzed by Two-Way Analysis of Variance of Treatment*Time (F = 5.83, P = 0.0.04). Tukey's pair-wise comparisons p<0.05 revealed significant difference in root biomass between control and warmed plots after the warming treatment started. Mean +/- S.E.

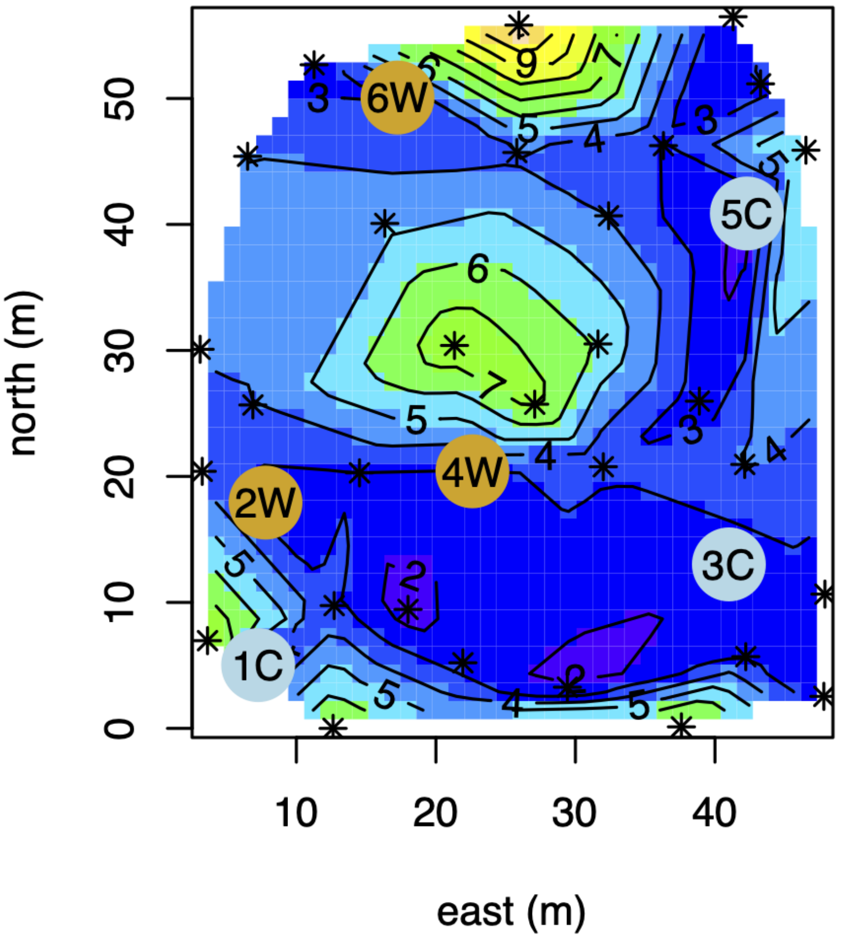

Supplemental Figure S2. Spatial interpolation of the mean soil CO_2 fluxes across the study site. Supplemental Figure 2 shows a spatial interpolation of the mean soil CO_2 fluxes, compared to the location of the long-term auto-sampling chambers. Asterisks indicate location of the 30 randomly distributed soil flux collars used to conduct the field spatial survey. In-figure numbers are respiration rates in micromols/m2/s, east and west axes are in meters, plot locations in blue are control plots (numbered with C) and plot locations in orange are warmed plots (numbered with W). There is significant spatial variability in the mean fluxes, indicating likely ‘hot’ and ‘cold’ spots for respiration. Furthermore, chamber 6 is on the edge of a hot spot, which may help to explain the extreme fluxes seen in that location.

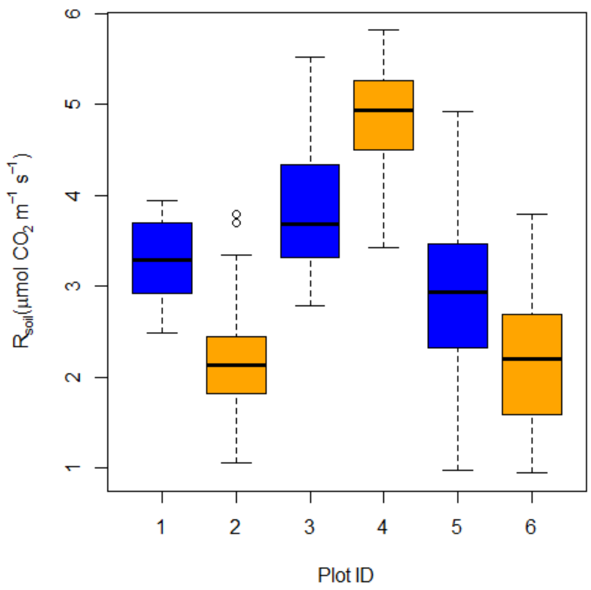

Supplemental Figure S3. Pre-warming soil respiration rates by plot. Box plot of pre-treatment soil respiration rates for all six plots. Odd numbers are control plots (blue), even numbers are warmed plots (orange). Difference between treatments was analyzed by Two-way Analysis of Variance (n=159; df = 1, F = 1.41, P=0.237).

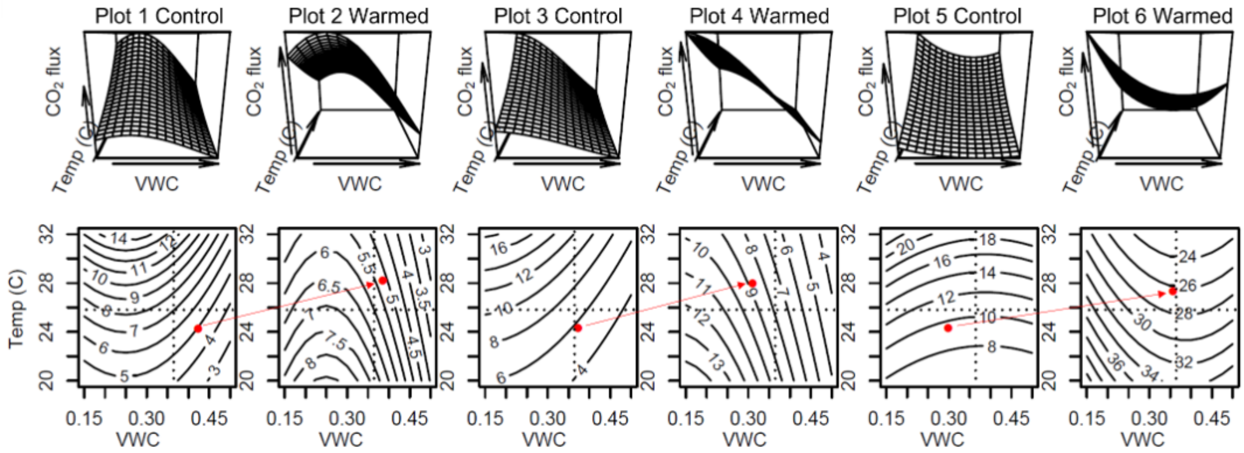

Supplemental Figure S4. Interactive effects of Soil moisture and Temperature on Soil Respiration. The upper row of figures shows the interactive effects of soil temperature and water content on soil CO2 efflux, as three-dimensional plots (x axis = volumetric water content; y axis = soil CO2 efflux and z axis = soil temperature). The lower row shows the contour plot representation of the same, with the x-axis showing volumetric water content, the y-axis showing soil temperature, and the contour lines representing modeled CO2 efflux at each combination of temperature and water content. The red dots are modeled CO2 flux for the mean temperature and water content of the plot. The red arrows show the change in mean temperature, moisture and resulting modeled change in CO2 efflux.

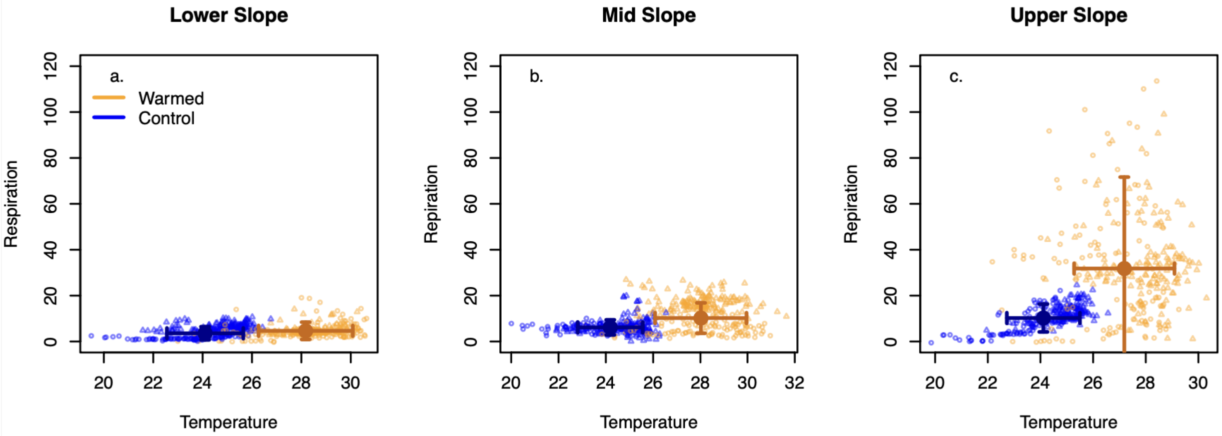

Supplemental Figure S5. Diurnal mean temperature versus diurnal mean respiration rate by topographic position and treatment presented on the same scale. Control (blue) and warmed (red) observations across the study period in the (a) Lower, (b) Mid, and (c) Upper slope paired plots. Error bars indicate standard deviation.

Supplemental Tables

	F-statistic (p-value) - all plots	F-statistic (p-value) - excluding upper slope plots with extreme values
Warming	4.99 (0.026)	**22.57(<0.0001)**
**Soil Moisture**	**184 (<0.0001)**	**173.62 (<0.0001)**
Soil Temperature	1.29 (0.26)	0.262 (0.609)
**Location**	**49.5 (<0.0001)**	**28.3 (<0.0001)**
**Warming x Location**	**11.92 (<0.0001)**	**4.35 (0.037)**
**Warming x Soil Temperature**	**23.8 (<0.0001)**	**8.63 (0.0034)**
**Soil Moisture x location**	**8.5 (<0.0001)**	**12.06 (0.0005)**

Supplemental Table S1. Generalized least squares model regression coefficients; model results with and without upper slope plots with extreme values. We present the ANOVA results from the best fit GLS model predicting soil respiration rates. Column 2, "All plots," uses the full dataset, while Column 3, "Excluding upper slope plots with extreme values," excludes chambers 5 and 6, which had very high values previously unobserved in any ecosystem we are aware of. While we do not have reason to exclude those values based on careful evaluation of the methods and the data, it is nonetheless instructive to verify that the results are robust to the inclusion or exclusion of those chambers.

coefficient	Value	Std.Error	t-value	p-value
(Intercept)	-0.485	0.542	-0.895	0.371
**warmed**	**3.088**	**0.732**	**4.217**	**0.000**
**soil temperature**	**0.092**	**0.021**	**4.493**	**0.000**
**vwc**	**-9.595**	**1.304**	**-7.360**	**0.000**
**vwc^2**	**-6.070**	**0.903**	**-6.725**	**0.000**
mid-slope	0.048	0.156	0.309	0.757
**upper slope**	**0.518**	**0.138**	**3.763**	**0.000**
**soil temp x warmed**	**-0.126**	**0.026**	**-4.882**	**0.000**
warmed x mid-slope	0.318	0.198	1.601	0.110
**warmed x upper slope**	**1.125**	**0.284**	**3.961**	**0.000**
**vwc x mid-slope**	**-6.012**	**1.804**	**-3.333**	**0.001**
**vwc x upper slope**	**6.813**	**2.665**	**2.557**	**0.011**
vwc^2 x mid-slope	2.350	1.293	1.817	0.069
**vwc^2 x upper slope**	**8.200**	**1.748**	**4.691**	**0.000**

Supplemental Table S2. Generalized least squares best model regression coefficients (for model details, see Supplemental Materials and Methods, below), using full dataset.

Supplemental Materials and Methods

A. Instrument Configuration and Data Processing

We used the LiCor LI-8100 + 8150 automated soil respiration system, which was connected to six long-term chambers (one chamber per plot). Excepting plot 4, which used a single 15 m cable, all chambers were connected using two 15 m cable extensions, for a total volume of 474 cm^3 for plots 1-3, 5-6 and 237 cm^3 for plot 4. Sample length was a total of 2 minutes with a deadband length of 25 seconds, a Pre-purge of 45 seconds, and a Post-purge of 45 seconds. Data were initially processed in Soil Flux Pro and transformed using plot specific hourly soil temperature data to calculate the flux due to consistent malfunction of the internal thermocouples of the individual chambers. Since temperature is converted to Kelvin, the calculations are relatively insensitive to temperature changes, with just 1% change in flux values with temperature differences of 3 ^oC. In addition, due to a zeroing of the humidity sensor in the instrument, we established a "Water Content Adjustment Procedure". We used a regression model to adjust water content values with site level % humidity data from 2017 through 2020. The observations were paired by day and hour. The relationship between water content observations was highly significant ($p < 0.005$). Using the intercept and slope coefficients from the regression model (25.05 and 1.09, respectively), we adjusted all raw water content values from March 2017, when the humidity sensor was zeroed, through September 5, 2017 when the system was turned off in preparation for Hurricane Irma, which ultimately struck the island causing island-wide loss of power. We used the equation:

$Z = a \times (X + b)$ Where: Z = adjusted H_2O values, X = raw H_2O values, a = slope, b = intercept

Once we adjusted all water content values, we recalculated the CO_2 flux values using the adjusted water content values and temperature data from soil temperature sensors. This correction led to minor adjustment of flux values.

B. Validating notably high soil CO2 fluxes

Many studies that utilize automated chambers exclude such extreme values from analyses; however, the exclusion of measurements based solely on the extremeness rather than the quality of the measurement itself (e.g., due to improper chamber closure), can result in data that are skewed to fit normal patterns and thus miss the potentially important contribution of "hot moments". Studies that use less frequent data collection also have the potential to miss these important contributions, because these elevated rates were so ephemeral.

We investigated whether the upper location and the hot moments that we observed were the result of alternate sources of CO2 (e.g., animal respiration from lizards, frogs, etc. trapped in the soil chamber during measurements) or that they could be due to equipment malfunction. As such, we invested considerable effort into validating our data. We describe the series of tests that we undertook below:

1. At a moment when one of the plots was exhibiting higher than expected fluxes, we tested the full factorial of equipment configurations to compare a plot that was demonstrating high fluxes with a plot that was demonstrating low fluxes:
    a. Switched the chamber that exhibited high fluxes with a chamber that exhibited low fluxes
    b. Changed the cable of the site with high fluxes and that of low fluxes

c. Used a different port under the above configurations to determine if the port was the issue.
d. Changed out all filters and checked all tubing.

Regardless of the configuration we used (i.e., new chamber, new cable, different port) the fluxes remained high in the high flux site and low in the low flux site.

2. In addition to testing the configuration, we also flushed all cables with 70% isopropyl alcohol per LiCor recommendation, to flush out any mold or insects that might have entered the cables that could be contributing to higher fluxes. Even after flushing cables, we continued to observe high fluxes.

3. One of the "malfunctions" that can occur with automated chambers is a failure to seal or close completely. We can identify these values by looking at the coefficient of variation of the individual measurements. As such, when we have a CV that is greater than 2% we look at the raw data and can evaluate whether the CO2 measurements increase linearly as we would expect. When this is not the case, we excluded the data.

4. Animals could also contribute to the high fluxes observed. We visually inspected all installed chambers and collars during the period of high fluxes and did not observe any frogs, lizards, ants, or other organisms that could contribute to the high fluxes observed. We additionally conducted tests where we manually blew CO2 into the LiCor IRGA and even with this test, we could not accomplish the high fluxes that we observed in the field. Given that we have not observed animals in the collars, and the fact that an adult male breathing directly into the instrument did not achieve the values observed, we concluded that it is highly unlikely the fluxes were driven by the small organisms that are found in our study area (frogs, lizards, snails – mongoose are the only mammal occasionally observed in our site), even temporarily. We also inspect the collars for branches that could impede closure and organisms as part of our weekly maintenance and trim seedlings that could also interfere with CO2 measurements.

5. As an additional confirmation that our data were valid, we evaluated the background CO2 to determine if these values were within the expected range. We examined raw data to see that from the moment measurements were made that the CO2 increased linearly within the chamber as would be expected, and we compared the range of raw CO2 data versus range of dry CO2 and compared the results with a different time frame which allowed us to confirm that the high fluxes were not the result of a calibration error. Further, we consulted LiCor scientists and upon presenting all evidence described above, they concurred that the high fluxes we were observed were indeed real.

C. Generalized least squares / linear mixed effects models

Using diurnal averaged soil respiration, temperature and water content, we evaluated treatment effects on soil respiration using a mixed modeling approach implemented in the 'nlme' package in R[1]. We log transformed the respiration rate and used the 2nd order polynomial fit of soil moisture, based on *a priori* expectations of the temperature and moisture responses of soil respiration[2]. We evaluated both linear

mixed effects, with chamber random effects, and generalized least squares model with different variance structures and approaches to temporal autocorrelation within a chamber. Model selection was conducted using Bayesian Information Criteria (BIC) to determine the variance and autocorrelation structure for repeated measurements within a chamber across time. After the best variance structure was found, backward selection was applied (using BIC) to determine the best set of fixed effects to include in the model. Parameter estimates for fixed effects (including warming treatment level, soil moisture, soil temperature, and hillslope location) were based on the subset of parameters and interactions that were significant in the best fit model. The best model was fitted via generalized least squares and accounted for autocorrelation of fluxes across days within a chamber as a first order autoregressive process with an estimated phi = 0.78, where variance was estimated as a function of measurement temperature and was allowed to vary between chambers[3]. The best model excluded interactions between soil temperature and hillslope location, as well as the interaction between warming treatment and soil moisture, but retained the main effects of each of these as well as the interaction between hillslope location and vwc, and warming treatment and soil temperature.

Full model: fit1 <- gls(log(flux) ~ treatment * location * poly(vwc,2) * temp, weights = varPower(form=~ temp|chamber), corr=corARMA(p=1, form=~ day|chamber), data=day.dat)

Best model: fit2 <- gls(log(flux) ~ treatment * location + poly(vwc,2) + poly(vwc,2):location + temp + treatment:temp, weights = varPower(form=~ temp|chamber), corr=corARMA(p=1, form=~ day|chamber), data=day.dat)

BIC fit1 = 768.4 BIC fit 2 = 652.7

In this model, the warming treatment significantly increased soil respiration. Moreover, soil respiration increased with soil temperature within the unwarmed treatment, but not the warmed treatments, and overall soil temperature was not a significant predictor across treatment groups. Overall, soil respiration decreased as soil moisture increased, this effect was only detectable at the lower and mid-slope locations. Soil respiration was highest at the upper slope location, and the magnitude of the warming effect on soil respiration was highest in the upper slope.

NOTE: The most obvious linear mixed effects model (with a chamber random effect and AR1 autocorrelation by day within chamber) was not possible because of model singularity. We would have to exclude the location variable or the chamber random effect. Accounting for autocorrelation by day within chamber via generalized least squares was the best model structure available.

Best model ANOVA

	F-statistic (p-value)
**Warming**	**4.99 (0.026)**
**Soil Moisture**	**184 (<0.0001)**
Soil Temperature	1.29 (0.26)
**Location**	**49.5 (<0.0001)**
**Warming x Location**	**11.92 (<0.0001)**
**Warming x Soil Temperature**	**23.8 (<0.0001)**

**Soil Moisture x location**	**8.5 (<0.0001)**

Best Model regression coefficients

coefficient	Value	Std.Error	t-value	p-value
(Intercept)	-0.485	0.542	-0.895	0.371
**warmed**	**3.088**	**0.732**	**4.217**	**0.000**
**soil temperature**	**0.092**	**0.021**	**4.493**	**0.000**
**vwc**	**-9.595**	**1.304**	**-7.360**	**0.000**
**vwc^2**	**-6.070**	**0.903**	**-6.725**	**0.000**
mid-slope	0.048	0.156	0.309	0.757
**upper slope**	**0.518**	**0.138**	**3.763**	**0.000**
**soil temp x warmed**	**-0.126**	**0.026**	**-4.882**	**0.000**
warmed x mid-slope	0.318	0.198	1.601	0.110
**warmed x upper slope**	**1.125**	**0.284**	**3.961**	**0.000**
**vwc x mid-slope**	**-6.012**	**1.804**	**-3.333**	**0.001**
**vwc x upper slope**	**6.813**	**2.665**	**2.557**	**0.011**
vwc^2 x mid-slope	2.350	1.293	1.817	0.069
**vwc^2 x upper slope**	**8.200**	**1.748**	**4.691**	**0.000**

Generalized least squares excluding upper slope chambers with extreme values

This analysis uses the same model (fit2) as above, excluding chambers 5 and 6, which had very high values previously unobserved in any ecosystem we are aware of. While we do not have reason to exclude those values based on careful evaluation of the methods and the data, it is nonetheless instructive to verify that the results are robust to the inclusion or exclusion of those chambers.

	F-statistic (p-value)
**Warming**	**22.57(<0.0001)**
**Soil Moisture**	**173.62 (<0.0001)**
Soil Temperature	0.262 (0.609)
**Location**	**28.3 (<0.0001)**
**Warming x Location**	**4.35 (0.037)**
**Warming x Soil Temperature**	**8.63 (0.0034)**
**Soil Moisture x location**	**12.06 (0.0005)**

Any use of trade, firm, or product names is for descriptive purposes only and does not imply endorsement by the U.S. Government

Supplementary References

1. Pinheiro J, Bates D, DebRoy S, Sarkar D, R Core Team (2019). nlme: Linear and Nonlinear Mixed Effects Models. R package version 3.1-139, <URL:https://CRAN.R-project.org/package=nlme>.

2. Davidson E.A., Janssens I.A. (2006). Temperature sensitivity of soil carbon decomposition and feedbacks to climate change. Nature. 440(7081):165-73.

3. Zuur A., Ieno E. N., Walker N., Saveliev A. A., & Smith G. M. (2009). *Mixed effects models and extensions in ecology with R*. Springer Science & Business Media.
